# How a physical exercise program performed by patients may impact caregiver burden in cancer: a qualitative study

**DOI:** 10.1007/s00520-025-10120-9

**Published:** 2025-11-08

**Authors:** Anita Borsati, Angela Marotta, Christian Ciurnelli, Francesco Bettariga, Gloria Adamoli, Lorenzo Belluomini, Federico Schena, Michele Milella, Cantor Tarperi, Robert U Newton, Sara Pilotto, Alice Avancini

**Affiliations:** 1 Biomedical, Clinical and Experimental Sciences, Department of Medicine, University of Verona, Verona, Italy; 2 Department of Neurosciences, Biomedicine and Movement, University of Verona, Verona, Italy; 3 Exercise Medicine Research Institute, Edith Cowan, University, Perth, Perth, Australia; 4 School of Medical and Health Sciences, Edith Cowan, University, Perth, Perth, Australia; 5 Section of Innovation Biomedicine-Oncology Area, Department of Engineering for Innovation Medicine (DIMI), University of Verona and University and Hospital Trust (AOUI) of Verona, Verona, Italy; 6 School of Human Movement and Nutrition Sciences, University of Queensland, Brisbane, Brisbane, Australia

**Keywords:** Physical exercise, Metastatic cancer, Caregivers, Caregiver’s burden

## Abstract

**Purpose:**

Caregivers of patients with metastatic cancer may be exposed to an overwhelming sense of burden. Physical exercise may help patients improve their physical condition, manage symptoms, and enhance their quality of life. Nevertheless, it is unclear if these interventions may also likely affect caregivers.

**Methods:**

Five focus group (*n* = 20 participants) were conducted to explore the caregivers’ experiences/perspectives of patients affected by metastatic cancer and performing a supervised physical exercise intervention. Thematic analysis using an inductive approach was performed. Theme, sub-themes, and illustrative quotes are displayed.

**Results:**

Four themes were identified. Theme 1 captured, with two sub-themes, the impact of the diagnosis on caregivers’ emotional status and daily routines. Theme 2 was related to the perceived benefits of patients’ engagement in the physical exercise program, enclosing three sub-themes (physical advantages, management of side effects, psychological well-being) and how this has helped caregivers enhance their emotional well-being. Theme 3 reported how caregivers have felt supported by the physical exercise program for the care of their loved ones (three-sub-themes: supervised by specialists, tailored and flexible program). Finally, theme 4 explained that caregivers, thanks to the patient’s participation in the exercise, were able to take back control of their own lives, having more time available and enhancing their relationship with the patient.

**Conclusion:**

Participation of patients with metastatic cancer in a structured physical exercise program may be effective in reducing caregiver’s burden. This study may serve as a trailblazer to guide future investigations and consolidate the present findings.

**Supplementary Information:**

The online version contains supplementary material available at 10.1007/s00520-025-10120-9.

## Introduction

Cancer can be a devastating disease not only for the patients themselves but also for the family members and friends, especially when the patient’s general health begins to deteriorate and they subsequently become unable to perform normal activities of daily living [1]. This condition forces family members/friends to become informal caregivers, i.e., an unpaid person who provides support to different services, e.g., administering medications, assisting patients in their activities, and managing finances [2]. Caregiving can be a demanding job since cancer caregivers spend an average of 33 h per week providing care [3], with consequential numerous limitations in their usual life, for example, the inability to leave home for long periods, enjoy usual entertainment, and keep up with work commitments [4]. Due to these major changes in their lifestyle—coupled with the psychological and physical changes of their beloved partners and family members—caregivers may experience an overwhelming sense of burden, leading very frequently to depression, isolation, financial constraints, and ultimately a decline in their overall health status as well [5]. Several factors may influence the level of caregivers’ burden, including demographic and personal traits (e.g., gender, education, anxiety) and features related to the patient [5]. For instance, the increased patient dependency level and the advanced stage of the disease are related to a more elevated burden [5].

A systematic review involving 6023 informal caregivers of patients with pancreatic cancer found that a substantial proportion reported clinically significant levels of anxiety and depression, and the primary sources of burden were related to the management of cancer-related symptoms and treatment side effects [6]. Moreover, the caregivers’ psychological well-being appears to be closely intertwined with that of the patients. In this sense, a strong correlation between anxiety and depression levels in patients and those of their caregivers was reported, highlighting the bidirectional nature of distress within the caregiving dyad [7]. This underscores the importance of supportive strategies that address both dyad members, as enhancing the patient’s well-being may lead to positive outcomes for the caregiver and vice versa.

In the advanced/metastatic cancer setting, physical exercise has been shown to be feasible and tolerable supportive care [8]. Physical exercise may be an optimal strategy to counteract the psycho-physical decline and the burdensome symptoms associated with the disease at an advanced stage. From a physical point of view, exercise has been demonstrated to increase the overall patient’s exercise tolerance by improving cardiorespiratory fitness, muscle strength, and body composition [9]. Moreover, participating in a physical exercise program may help to optimize the quality of life and manage different symptoms, such as insomnia, dyspnea, feelings of anxiety, and depression [9]. A recent randomized controlled trial conducted on 357 patients affected by metastatic breast cancer has demonstrated that a 9-month supervised physical exercise program, including aerobic, resistance, and balance activities, produced a significant positive impact on overall quality of life and fatigue levels [10].

While much attention has been given to the direct effects of exercise on patient outcomes, less is known about the indirect benefits that such interventions may have on caregivers. Most caregiver-focused interventions currently rely on psychoeducation, self-management, or communication training aimed at addressing unmet needs. However, the evidence supporting the effectiveness of these interventions is still limited [11], and many of them risk adding further responsibilities to already overburdened individuals, without necessarily addressing the patient’s health needs, which often represent the primary source of caregiver stress [12, 13]. Emerging evidence also shows that the physical and emotional challenges associated with caregiving can lead to the adoption of unhealthy behaviours, including physical inactivity [14]. A recent review examining exercise interventions for caregivers across various diseases found that only about half of the studies reported improvements in psychological outcomes such as anxiety, stress, and depression, and none showed a significant reduction in caregiver burden [15]. A common barrier identified was lack of time, highlighting the need for supportive strategies that do not add further demands but can be integrated into the existing routines of caregiving families [16]. Despite this, to our knowledge, no previous studies have investigated the indirect impact of a structured physical exercise intervention for patients with metastatic cancer on their informal caregivers. Our hypothesis is that interventions aimed at patients, even if not specifically designed for caregivers, may have a ripple effect on those who provide informal care. Therefore, the aim of this study was to explore the experiences and perspectives of informal caregivers whose loved ones, affected by metastatic cancer, participated in a supervised physical exercise program.

## Methods

### Study design and participants

The Consolidated Criteria for Reporting Qualitative Research (COREQ) guidelines for qualitative research [17] were followed to report the study. A purposive sampling method was used to recruit informal caregivers of patients affected by metastatic cancer who performed a supervised physical exercise program previously detailed and tested [18], and which currently is offered to patients. Briefly, the program consists of 3-month, bi-weekly sessions of combined aerobic and resistance training supervised by exercise professionals with experience in cancer and delivered through different modalities, i.e., home-based or at the facilities, based on patients’ preferences. Each program is individually tailored to the patient’s baseline assessments and comprises aerobic components performed at moderate intensity for 10–30 min per session with duration progression over the weeks. Resistance training includes six exercises performed with body weight or elastic bands for 2–3 sets of 8–12 repetitions, which progressively increase at moderate intensity.

Participants were recruited by the research team at the Department of Neuroscience, Biomedicine, and Movement Sciences, University of Verona. Ethical approval for the study was obtained from the Ethics Committee of the University of Verona (Protocol: CARP #04/2024), and all participants signed the informed consent. Sampling and data collection continued until saturation was achieved, ensuring no new information emerged from additional interviews.

### Data collection and setting

Five focus groups were organized either in person (*n* = 3) or online via Zoom platform (*n* = 2) [19], during the “Run for Science” (R4S) project. R4S is a research project, annually organized by the University of Verona, which involves several scientific institutions worldwide to explore the impact of physical activity [20]. Eligible caregivers were contacted in advance by the research team and invited to join R4S as study participants. During the event, caregivers who consented to participate were grouped into focus groups, which were conducted in a quiet, reserved room on-site to ensure a comfortable and private environment for discussion. All in-person interviews were moderated by a clinical psychologist (A.M.) with the assistance of two PhD students trained in qualitative research (A.B. and C.C.). For those who were unable to attend the event or preferred a remote option, online focus groups were scheduled in the following days via Zoom. These online sessions followed the same format and were conducted with the same team to maintain consistency.

Focus groups followed a semi-structured format using an interview guide developed by a multidisciplinary team (kinesiologists, psychologists, oncologists). The interview guide (Supplementary Information) was based on existing literature and aimed to explore caregivers’ perceptions regarding the impact of the structured physical exercise program. Although participants were asked about perceived changes in their loved ones, the primary focus of the interviews was to explore the caregivers’ own experiences, particularly in terms of physical, psychological, and social or family-related consequences. Specifically, the aim was to understand whether the program may have alleviated caregiver burden. The open-ended questions allowed the facilitator to probe deeper when relevant topics emerged spontaneously. Data collection continued until data saturation was achieved, defined as the point at which no new information or themes emerged in subsequent focus groups. After conducting five sessions, the research team reached consensus that thematic saturation had been reached, and no additional sessions were required. Caregivers’ sociodemographic details (birth date, education level, marital status, occupational status, family income, and type of relationship with the patient) and their current exercise level assessed using the Godin Leisure-Time Exercise Questionnaire as well as the patient’s clinical information (cancer type and stage, diagnosis date and treatment status) were self-reported using a dedicated questionnaire (Supplementary Information). Interviews lasted 45–60 min, were audio-recorded, and were transcribed verbatim.

### Analysis

Focus groups were analyzed using the reflexive inductive thematic analysis and the Atlas.ti™ software. The reflexive inductive process was chosen because of its flexibility and the potential to offer a rich understanding of participants’ experiences. After multiple readings of the transcripts to capture meaningful content aligned with the study’s research questions, the researchers developed a preliminary list of codes. These codes were then refined through group discussions, grouped into categories, and themes constructed. Each focus group was independently analyzed by A.B. and F.B.; codes, categories, and themes were then discussed with the coding authors and A.A. to resolve disagreements and further refine the final themes and sub-themes. The four quality criteria to enhance the trustworthiness of the research were applied: credibility, confirmability, dependability, and transferability. Credibility was reinforced by engaging over an extended period with participants, which helped to actively interpret and not merely describe the interviews. To ensure confirmability and maintain a more objective stance, three experienced researchers (A.B., F.B., A.A.) independently coded the transcripts. Peer debriefing and collaboration with a multidisciplinary team, including kinesiologists, psychologists, and oncologists, was employed to mitigate researcher bias and offer validated findings. Dependability was achieved via a detailed audit trail of research decisions, including changes in methodologies or analysis, promoting transparency of the study. Lastly, transferability was supported by detailed descriptions of the study context and sampling methods, enabling application in similar settings.

## Results

Twenty caregivers agreed to participate in the study. Caregivers were mainly female (70%), had a mean age of 58.9 years, and were principally spouses or partners (80%) of patients who were affected by mixed cancer types at advanced/metastatic stage and currently undergoing anticancer treatments. Socio-demographic characteristics of the participants is presented in Table 1.

**Table 1:** Characteristics of the study participants

Characteristics (*n* = 20)	*N* (%)
**Age in years**	59.25 ± 12.82
**Gender**	
Male	6 (30)
Female	14 (70)
**Education**	
Secondary	6 (30)
High school degree	8 (40)
Undergraduate degree	4 (20)
Postgraduate degree	2 (10)
**Marital status**	
Single	1 (5)
Married	18 (90)
Widow	1 (5)
**Employment**	
Full-time employed	5 (25)
Part-time employed	3 (15)
Retired	10 (50)
Stay-at-home-parent	2 (10)
**Family income**	
More than adequate	3 (15)
Adequate	16 (80)
Barely adequate	1 (5)
**Degree of kinship**	
Wife/husband	17 (85)
Sibling	2 (10)
Offspring	1 (5)

### Conceptual framework overview

**Fig. 1:**
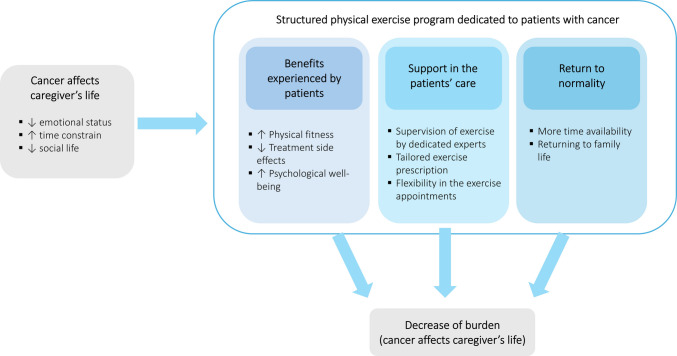
Conceptual framework associating physical exercise performed by patients with caregivers’ burden

The conceptual framework that emerged from our analysis is displayed in Fig. 1. It captures a progressive process in which caregivers experience a shift from emotional and practical distress toward improved well-being and reduced burden, facilitated by their loved ones’ participation in a structured physical exercise program. Participants stated that cancer diagnosis strongly affected them emotionally but also interrupted their daily routine and relationship with their loved ones. These aspects represent the initial condition of vulnerability and stress, which shapes how caregivers perceive subsequent changes. The structured exercise program introduced a series of interconnected benefits. Caregivers observed noticeable improvements in their loved ones’ physical functioning, symptom control, and psychological well-being. These visible changes acted as a source of emotional relief for the caregivers, reducing their sense of helplessness and anxiety. Witnessing these improvements contributed to a more optimistic view of the illness and strengthened their own resilience. The structure and professional supervision of the program were perceived as critical in reducing the caregiver’s sense of responsibility and isolation. Caregivers emphasized that having dedicated exercise experts monitor the patient’s health and personalize the program based on their clinical status generated a sense of reassurance and shared responsibility in the care process. The logistical flexibility of the sessions, designed to align with hospital appointments, further eased the organizational burden. As patients became more autonomous and emotionally stable, caregivers reported regaining time for self-care and the ability to resume neglected routines, hobbies, and relationships. This return to normality included both individual activities and the rediscovery of positive moments shared with their loved ones, contributing to a renewed sense of family balance and connection. The process culminates in a reduction of perceived caregiver burden. Illustrative quotes for each framework element are shown in Table 2.

**Table 2:** Key findings and illustrative quotes emerged from the focus groups

Themes and subthemes	Illustrative quotes
**The cancer diagnosis affects the caregiver’s life**	
The negative impact on emotional status	“When the diagnosis first came, it brought a profound sense of fear” (Caregiver 13, focus group 3) “There’s this feeling of exclusion that causes real emotional pain, not because someone explicitly says, ‘You can’t understand,’ but because it’s subtly shown through actions or attitudes. For those who are supporting someone facing the illness, this unspoken dynamic can be incredibly frustrating” (Caregiver 16, focus group 4)
The negative impact on patient-caregiver relationship and disruption of normal life	“In the past, we could occasionally share activities together, like going for a jog. Now, that’s something that’s simply no longer possible. Initially, this change only served to amplify the sense of distance between us. We both struggled with it, but I think I felt the weight of it even more” (Caregiver 3, focus group 1) “Even something as simple as making plans, thinking about what to do, where to go, or how to spend a Sunday, has become more challenging. Decisions that used to be straightforward now feel uncertain because everything depends on how the other person feels that day. It’s not just big plans, but also everyday activities that have become more limited” (Caregiver 11, focus group 3)
**The benefits experienced by patients have also stronger the caregivers**	
Improvements in physical fitness	“My wife had significant difficulty in walking, but since she started this program, everything has changed. She essentially learned how to walk again, thanks to the guidance and support she received. It’s been incredibly important for her” (Caregiver 2, focus group 1) “Physically, I’ve noticed improvements in his posture, balance, and overall strength. Even tasks like picking up objects feel much easier and more fluid now” (Caregiver 9, focus group 2)
Reduction of treatment-related side effects	“At the beginning, he faced many challenges. He had lost considerable weight because the therapy made eating very difficult, and as a result, he also lost muscle mass. However, after starting the exercise program, he began to recover both his strength and his normal weight” (Caregiver 5, focus group 1) “From a physical point of view, the therapy had really weakened her. She had neuropathy in her hands and feet, which made everyday tasks like driving very difficult. Through this program, she’s experienced noticeable improvements in muscle function and in sensitivity” (Caregiver 13, focus group 3) “After completing the first-line therapies, she had to start a hormone therapy that caused side effects. This activity program helped her counteract those effects, such as stiffness, and allowed her to regain muscle mass and mobility” (Caregiver 11, focus group 3)
Improvements in psychological well-being	“The motivation and self-esteem he gained are absolutely fundamental from a psychological perspective. They play a key role in helping him face this challenging time” (Caregiver 4, focus group 1) “I’ve noticed a significant improvement in his mood and a reduced tendency to isolate from others” (Caregiver 16, focus group 4)
**Feeling supported in the patient’s care**	
Supervision of exercise by dedicated experts	“If you have any doubts, you can always reach out to your personal trainer. They’re consistently available and in touch, which gives a reassuring sense of support. The great thing is that we’re never left on our own; there is always someone to help if something isn’t right” (Caregiver 15, focus group 4) “He had the opportunity to exercise with some truly skilled, knowledgeable, and well-prepared exercise professionals. Most importantly, they teach him valuable things like how to walk, how to move, and how to manage pain and I’ve never seen him get hurt. This support also gives me peace of mind, knowing he’s in good hands” (Caregiver 1, focus group 1)
Tailored exercise prescription	“When we discovered the exercise program, I felt like a weight had been lifted off my shoulders. I thought, ‘Ah, they’ll take care of this.’ It was such a relief for me because I realized that, on my own, I would never have been able to do something like this” (Caregiver 3, focus group 1) “It makes me feel good to see him more confident, knowing he’s being followed closely and being part of a dedicated program. This has been incredibly important to me from the very beginning. The entire team working in the oncology unit created a kind of protective circle around us. This made us feel cared for and supported in a way that truly mattered” (Caregiver 7, focus group 2)
Flexibility in exercise appointments	“He felt really upset if he had a medical appointment that made him miss an exercise session. Having the option to reschedule was such a relief, it made things so much easier and helped him stay committed” (Caregiver 1, focus group 1)
**Taking back control of own life**	
Having more time availability	“I felt a sense of freedom… the fact that he could train on his own elsewhere allowed me to focus on myself, to meet up with a friend and reconnect with my social life. During those hours, my mind could take a break from the constant worry of the disease, allowing me to return with more serenity and acceptance” (Caregiver 9, focus group 2) “The fact that he’s feeling better and is more physically independent has allowed me to regain some independence, too. For example, I can now go out for a walk on my own, something I didn’t feel comfortable doing before. Being able to do that again has been uplifting for me” (Caregiver 15, focus group 4)
Returning to family life	“The exercise program has significantly improved our lives. It feels like we’ve gone back to how we were before, with a renewed energy and desire to be active. We’ve always been like this, but with the illness, those things were lost. After chemo, he often had to stay in bed, feeling unwell, and those moments of vitality seemed far away” (Caregiver 19, focus group 5) “We’ve also started sharing more experiences. For instance, we’ve gone out for lunch a few times, something that rarely happened before. Previously, our lives as a couple were entirely centered on the illness and its treatments. Now, things feel lighter, more serene, and less weighed down. This newfound balance has been beneficial for both of us” (Caregiver 17, focus group 4)

### The cancer diagnosis affects the caregiver’s life

Two relevant sub-themes were identified within this theme. (1) The first was related to the negative impact of a cancer diagnosis and its physical consequences on the caregiver’s emotional status. The fear of the disease and the associated negative beliefs of patients were often mirrored by caregivers: “It was truly emotionally devastating; within just three months, she could no longer walk, which became a huge challenge for us” (Caregiver 2, focus group 1). Additionally, caregivers also highlighted how their own sufferings and needs often went unnoticed, as attention was primarily focused on the patient: “Everyone asked me how my wife was doing, but no one asked how I was, even though I was suffering as well” (Caregiver 3, focus group 1). (2) The second sub-theme referred to the patient-caregiver relationship. Caregivers reported that the burden of cancer and treatments also strained their relationship with the patient, often causing alienation and interfering with normal daily activities and routines. They emphasized the difficulty of scheduling free-time activities due to the treatment-related side effects, as well as the limitation in their social life due to the time demanded by caregiving: “We live far from the hospital, and since he could not drive, I took him to his treatments…I only went out for essentials like grocery shopping or trips to the pharmacy; everything else revolved around being there for him” (Caregiver 14, focus group 3).

### The benefits experienced by patients also strengthened the caregivers

This theme was composed of three sub-themes: (1) the physical improvements, (2) the management of treatment-related side effects, and (3) the psychological well-being produced by physical exercise.

Caregivers expressed a wide variety of fitness improvements perceived in their loved ones related to physical exercise, including an increase in cardiorespiratory fitness, flexibility, muscle strength, and mass, an enhancement in posture, and easier performance of daily living activities. Engaging in a physical exercise program has been recognized as a strategy to ameliorate symptoms and side effects, especially fatigue level, sleep quality, peripheral neuropathy, and regaining normal weight after loss. From a psychological point of view, caregivers have noticed enhancements in patients’ moods, particularly a decrease in feelings of anxiety and isolation and an increase in happiness, socialization, and self-esteem, which have also been translated into a better approach to the disease. All these positive changes detected in their loved ones have been translated into a better perception of the cancer disease by the caregivers, reporting less feelings of worry and a major sense of “emotional lightness” associated with cancer: “Seeing the benefits of physical exercise on your wife, it gives you the strength to face this situation; it is like adding medicine to medicine” (Caregiver 2, focus group 1).

### Feeling supported in the patient’s care

Overall, the caregivers underlined how some characteristics of the physical exercise program have allowed them to feel supported in the patient’s care, as described in three sub-themes. (1) First, the supervision by dedicated exercise specialists who actively and constantly interacted with patients by asking how they felt and checking the exercise effort and compliance was perceived as a form of monitoring, thus mitigating the caregiver’s stress. (2) A second emerged sub-theme was the tailoring of the exercise prescription based on the patient’s needs and on cancer and treatment symptoms, which has been recognized as acting as a supportive and personalized approach, giving caregivers a feeling of being “not alone” on the cancer journey and a sense of reassurance: “I was worried because my mom did not have a structured plan, just some occasional walks; engaging in this program tailoring to her condition gave me peace of mind” (Caregiver 5, focus group 1). (3) The third aspect was the flexibility of the program, indicated as the possibility of scheduling or moving the programmed training based on hospital appointments so as not to overlap with them but also planning sessions on the same days, which has been reported as a smoothing option in the organization of the numerous patient’s commitments related to the disease.

### Taking back control of own life

Caregivers explained that patients’ participation in the physical exercise program had permitted them to re-perform routine activities in two sub-themes: (1) having more time availability and (2) returning to family life. In the first sub-theme, caregivers underlined the possibility of having more time for self-care, growing their friendships, and their hobbies. This “sense of freedom” expressed by the participants was primarily since patients spent at least 2 h per week in training, “I am happy when my husband comes to the gym because I have more time to do what I want” (Caregiver 16, focus group 4), and to the increased autonomy level of the patients, enabling them to manage one’s travels and daily activities, and thus allowing caregivers to focus more on their own lives. The second sub-theme is associated with an improved relationship with their loved ones by discovering unladen moments, a positive atmosphere at home, sharing activities, e.g., walking, going to restaurants together, and enhanced communication, also speaking of those topics, such as the illness itself, that was considered a “taboo” until then.

## Discussion

This qualitative analysis explored how the perceptions of physical exercise performed by patients affected by advanced or metastatic cancer impacted caregivers’ burden. Participants in this study recognized that cancer diagnosis had a tremendous impact on their lives, from a psychological point of view to social activities, but, at the same time, they highlighted how these impairments might be ameliorated by the participation of their loved ones in a tailored physical exercise program.

The concept that cancer affects not only patients but also their families or friends, thus becoming a full-fledged “family disease,” was discovered long ago. In our report, caregivers emphasized both the social and emotional impact of cancer on themselves, highlighting the worries about the disease, in particular, related to physical changes observed in the patients, the difficulties in having free time, or scheduling hobbies, often due to medical issues. Prior investigations have reported similar findings [21] and extend the caregivers’ challenges to financial, spiritual, and physical well-being [22]. In reality, the degree of caregiving may differ according to symptom burden, stage of disease, and type of anticancer treatment, but it usually is more intense in the treatment periods, taking up about 8.8 h per day, and could last up to 4 years [23, 24]. Taking these aspects together, caregivers of patients affected by metastatic disease are those who may be more exposed to experience impairments and spend more time in caregiving activities. Moreover, given the advances in terms of therapeutic options, the life span of patients with metastatic disease is improved, and it is expected to increase [25, 26]; therefore, it is possible to speculate that the overall length of caregiving will also be prolonged. From these perspectives, it is essential to offer strategies for supporting caregivers to ameliorate the burden that they may face. Although interventions directly targeting caregivers, such as educational programs or cognitive behavioral therapy, have been explored, they remain challenging to implement. Financial constraints, the additional responsibilities these interventions impose, and the predominant focus on the patient during treatment often limit their long-term impact. A recent meta-analysis further demonstrated that such interventions have limited efficacy in reducing caregiver burden, depression, or anxiety [27]. One hypothesis for this outcome is that these interventions often fail to account for the reciprocal nature of the patient-caregiver relationship. In this dynamic, the caregiver’s well-being influences, and is influenced by, the patient’s physical and psychological state [28, 29]. Our findings support this interplay: caregivers reported improvements in their mood and well-being when they observed positive changes in their loved one’s health. The caregiver burden is a multifaceted phenomenon encompassing emotional, physical, and social dimensions. Addressing only one of these aspects or focusing exclusively on the patient or the caregiver may not be sufficient. Our study offers a novel perspective by demonstrating that even without interventions specifically targeting caregivers, indirect benefits can emerge when patients participate in structured physical exercise programs.

Although we did not measure caregiver burden before and after the intervention, caregivers consistently reported feeling supported and experiencing relief. We speculate that these benefits arise because, compared to other supportive strategies, physical exercise can improve patients’ physical and psychological health, reduce symptoms, and enhance their autonomy, thereby decreasing their dependence on caregivers. Additionally, several aspects of the exercise program were appreciated by caregivers like the patient-centered focus, which indirectly made them feel supported and reassured that their loved one’s needs were being addressed comprehensively, the alignment of exercise sessions with hospital appointments minimized logistical challenges, reducing the likelihood of overloading caregivers with additional commitments, and the professional supervision during exercise sessions, which ensured that patients were monitored for their health and safety. This aspect alleviated some of the psychological burden associated with constant vigilance over the patient’s well-being. These findings underscore the potential of indirect interventions, such as structured exercise programs, to offer psychological and practical relief to caregivers.

Future research should build upon these findings through randomized controlled trials to evaluate and compare the effects of different intervention models dedicated to patients, caregivers, or both as a dyad on caregiver burden and quality of life. Such studies could help elucidate the most effective strategies to support informal caregivers while ensuring the sustainability of these interventions over time.

## Limitations

The major strength of this study is the novelty of the investigated topic and the inductive approach utilized. This study presents some limitations that may affect the interpretation and generalizability of the results. First, our sample was predominantly female and largely composed of individuals from middle to higher socioeconomic backgrounds. This demographic skew may introduce bias, as caregiving experiences and the perceived benefits of exercise can vary significantly across gender and socioeconomic strata. Importantly, exploring the perspectives of caregivers from lower socioeconomic backgrounds would be highly relevant, as caregiving is not only emotionally and physically demanding but can also pose a significant financial burden. In such contexts, even interventions with recognized benefits, such as structured physical exercise, must be evaluated in terms of their economic accessibility and sustainability. Future studies should investigate whether and how exercise interventions can be adapted to remain beneficial yet affordable and feasible for families with limited financial resources. Moreover, a potential selection bias may have influenced participation, as individuals more interested or favourably inclined toward physical exercise might have been more motivated to join the study. Finally, although caregivers consistently reported perceived improvements, the study did not include pre- and post-intervention assessments of caregiver burden, which limits the ability to draw causal inferences. Despite these limitations, our study offers a novel perspective and is grounded in a rigorous qualitative methodology. Interviews were conducted shortly after the exercise program to minimize recall bias, and a multidisciplinary research team supported the inductive thematic approach.

## Conclusion

Our findings suggest that a structured physical exercise program performed by patients with advanced cancer may have a beneficial indirect effect on their caregivers. Most participants reported that the improvements observed in their loved ones such as better physical function, mood, and autonomy, translated into enhanced psychological well-being and emotional relief for themselves. The program’s structured nature and professional supervision reassured caregivers, reduced their need for constant monitoring, and allowed them to reclaim time for self-care. These results support integrating exercise programs into oncology care not only to benefit patients but also as a potential indirect intervention for caregivers. Such programs should be as follows: (i) flexible and accessible, aligning with patients’ medical appointments and offering different delivery options to minimize caregiver burden; (ii) supervised by trained professionals to ensure safety and maximize patient engagement, easing caregiver stress; (iii) designed with the patient-caregiver dyad in mind, recognizing the close link between caregiver well-being and patient health; and (iv) accompanied by information and support for caregivers, involving them actively or keeping them informed. Future clinical interventions should consider developing dyad-based models where both patients and caregivers are supported in a coordinated manner. While the primary focus of the intervention may remain on the patient, recognizing and addressing the caregiver’s role and needs could enhance the overall impact and sustainability of such programs.

## Supplementary Information

Below is the link to the electronic supplementary material.

ESM1 (DOCX.24.2 KB)

## Data Availability

No datasets were generated or analysed during the current study.
