# Correction to: How a physical exercise program performed by patients may impact caregiver burden in cancer: a qualitative study

**DOI:** 10.1007/s00520-025-10253-x

**Published:** 2025-12-20

**Authors:** Anita Borsati, Angela Marotta, Christian Ciurnelli, Francesco Bettariga, Gloria Adamoli, Lorenzo Belluomini, Federico Schena, Michele Milella, Cantor Tarperi, Robert U. Newton, Sara Pilotto, Alice Avancini

**Affiliations:** 1 Biomedical, Clinical and Experimental Sciences, Department of Medicine, University of Verona, Verona, Italy; 2 Department of Neurosciences, Biomedicine and Movement, University of Verona, Verona, Italy; 3 Exercise Medicine Research Institute, Edith Cowan, University, Perth, Perth Australia; 4 School of Medical and Health Sciences, Edith Cowan, University, Perth, Perth Australia; 5 Section of Innovation Biomedicine‑Oncology Area, Department of Engineering for Innovation Medicine (DIMI), University of Verona and University and Hospital Trust (AOUI) of Verona, Verona, Italy; 6 School of Human Movement and Nutrition Sciences, University of Queensland, Brisbane, Brisbane Australia

**Correction to: Supportive Care in Cancer (2025) 33:1029**

10.1007/s00520-025-10120-9

On pages 3–4, there is a paragraph beginning with “Key findings and illustrative quotes emerged from the focus… ‘This newfound balance has been beneficial for both of us’ (Caregiver 17, focus group 4).” This paragraph should not appear in the main text, as the information is already included in Table 2.

The publisher regrets this error.

The original article has been corrected.

