# Supplementary material for: How a physical exercise program performed by patients may impact caregiver burden in cancer: a qualitative study
Source: Support Care Cancer. 2025 Nov 8;33(12):1029. doi: 10.1007/s00520-025-10120-9 (PMC12594653; doi:10.1007/s00520-025-10120-9)
Supplement: Supplementary file 1 — (DOCX.24.2 KB) [file 520_2025_10120_MOESM1_ESM.docx]

**Supplementary file 1**. Interview’s guide

| **Questions** |
| --- |
| 1. What are the benefits that you have observed for your loved one related to participation in the structured physical exercise program? |
| 1. Did you notice any positive changes, either in terms of physical, psychological, or social benefits from engaging in physical exercise? |
| 1. What are the disadvantages that you have observed for your loved one related to participation in the structured physical exercise program? |
| 1. Did you notice any negative changes, either in terms of physical, psychological, or social aspects related to physical exercise? |
| 1. What are the physical, psychological, and social effects, that you have observed on yourself from your loved one’s participation in the structured physical exercise program? |
| 1. How has your loved one’s participation in the structured physical exercise program affected you from a physical or psychological point of view? |
| 1. Caring for someone else can affect health, financial resources, and daily schedule. What changes have you observed in your daily activities due to your loved one’s participation in the structured physical exercise program? |
| 1. Have you noticed any changes in your mental/physical health, finances, family relationships, or self-esteem? |

**Supplementary file 2.** Questionnaire completed by caregivers

**Gender:**

- Male
- Female

**Date of birth:** _____/______/_______________

**What is your level of education?**

- Elementary school
- Middle school
- High school diploma
- University degree
- Postgraduate degree

**What is your marital status?**

- Single
- Married
- Divorced
- Widowed

**What is your current employment status?**

- Full-time employment
- Part-time employment
- Unemployed
- Retired
- Homemaker
- Other

**With your current financial resources, how easily do you make it to the end of the month?**

- Very easily
- Fairly easily
- With some difficulty
- With great difficulty

**What is your relationship with the patient?**

- Father/Mother
- Husband/Wife
- Son/Daughter
- Uncle/Aunt
- Cousin
- Brother/Sister

**What is the primary site of your loved one's cancer?**

- Colorectal
- Lung
- Breast
- Head and neck
- Upper gastrointestinal (pancreas, esophagus, stomach, duodenum, liver, bile ducts)
- Gynaecological (uterus, ovary, endometrium)
- Genitourinary (prostate, kidney, bladder, testicle)
- Melanoma
- Hematological (lymphoma, myeloma, leukemia)
- Other

**What treatments/therapies has your loved one undergone? (You may select more than one option)**

- Surgery
- Chemotherapy
- Radiotherapy
- Hormone therapy
- Bone marrow transplant
- Other

**In what month and year was your loved one’s cancer diagnosed?** ______/_______________

**What is the status of your loved one’s treatment (e.g., chemotherapy, biological therapy, immunotherapy, hormone therapy)?**

- Not yet started
- Currently ongoing
- Completed (no further treatments planned)
- Undefined/Don’t know

**In the past week, how many days and for how long did you engage in aerobic physical activity (e.g., walking, cycling, swimming) at an intensity where your heart rate and breathing were elevated above normal?**

Please indicate the number of days per week and the average duration (in minutes) per day.

Weekly frequency (n)____________ Mean duration per session (min.)_____________

**In the past week, how many days and for how long did you engage in physical activity to increase muscle strength, such as lifting weights?**

Please indicate the number of days per week and the average duration (in minutes) per day.

Weekly frequency (n)____________ Mean duration per session (min.)_____________

**During your leisure time in the past week (last 7 days), on average, how many times did you perform the following types of exercise for more than 15 minutes? Please indicate the number of times per week.**

HIGH-INTENSITY PHYSICAL ACTIVITY (This involves strenuous physical effort, significantly increases breathing, and makes speaking difficult during the activity.)

- 0
- 1
- 2
- 3
- 4
- 5
- 6
- 7
- 8
- 9
- 10

During your leisure time in the past week (last 7 days), how much total time did you spend on HIGH-INTENSITY PHYSICAL ACTIVITY in one day?

Please indicate the total hours and minutes for one day: _________________________________

**During your leisure time in the past week (last 7 days), on average, how many times did you perform the following types of exercise for more than 15 minutes? Please indicate the number of times per week.**

MODERATE-INTENSITY PHYSICAL ACTIVITY (This involves moderate physical effort, slightly increases breathing, and allows speaking with some difficulty.)

- 0
- 1
- 2
- 3
- 4
- 5
- 6
- 7
- 8
- 9
- 10

During your leisure time in the past week (last 7 days), how much total time did you spend on MODERATE-INTENSITY PHYSICAL ACTIVITY in one day?

Please indicate the total hours and minutes for one day: _________________________________

**During your leisure time in the past week (last 7 days), on average, how many times did you perform the following types of exercise for more than 15 minutes? Please indicate the number of times per week.**

LOW-INTENSITY PHYSICAL ACTIVITY (This involves minimal physical effort and allows comfortable speaking during the activity.)

- 0
- 1
- 2
- 3
- 4
- 5
- 6
- 7
- 8
- 9
- 10

During your leisure time in the past week (last 7 days), how much total time did you spend on LOW-INTENSITY PHYSICAL ACTIVITY in one day?

Please indicate the total hours and minutes for one day: _________________________________
